# An optimized imaging protocol for [^99m^Tc]Tc-DPD scintigraphy and SPECT/CT quantification in cardiac transthyretin (ATTR) amyloidosis

**DOI:** 10.1007/s12350-021-02715-6

**Published:** 2021-07-30

**Authors:** Imke Schatka, Anne Bingel, Franziska Schau, Stephanie Bluemel, Daniel R. Messroghli, David Frumkin, Fabian Knebel, Sonja M. Diekmann, Ahmed Elsanhoury, Carsten Tschöpe, Katrin Hahn, Holger Amthauer, Julian M. M. Rogasch, Christoph Wetz

**Affiliations:** 1 Department of Nuclear Medicine, Charité - Universitätsmedizin Berlin, Corporate Member of Freie Universität Berlin, Humboldt-Universität zu Berlin, and Berlin Institute of Health, Berlin, Germany; 2 Department of Internal Medicine and Cardiology, Deutsches Herzzentrum Berlin, Berlin, Germany; 3 DZHK (German Center for Cardiovascular Research), Partner Site Berlin, Berlin, Germany; 4 Medical Clinic for Cardiology, Angiology, Pneumology, Charité - Universitätsmedizin Berlin, Corporate Member of Freie Universität Berlin, Humboldt-Universität zu Berlin, and Berlin Institute of Health, Campus Charité Mitte (CCM), Berlin, Germany; 5 Department of Cardiology, Charité - Universitätsmedizin Berlin, corporate member of Freie Universität Berlin, Humboldt-Universität zu Berlin, and Berlin Institute of Health, Campus Virchow Klinikum (CVK), Berlin, Germany; 6 Berlin Institute of Health (BIH) Berlin-Brandenburger Center for Regenerative Therapies (BCRT), Charité, Berlin, Germany; 7 Department of Neurology, Charité - Universitätsmedizin Berlin, Corporate Member of Freie Universität Berlin, Humboldt-Universität zu Berlin, and Berlin Institute of Health, Berlin, Germany; 8 Berlin Institute of Health (BIH), Berlin, Germany

**Keywords:** Cardiac ATTR amyloidosis, DPD, bone scan, quantification, SPECT/CT, CZT, 90°, L-mode, H-mode

## Abstract

**Background:**

In [^99m^Tc]Tc-DPD scintigraphy for myocardial ATTR amyloidosis, planar images 3 hour p.i. and SPECT/CT acquisition in L-mode are recommended. This study investigated if earlier planar images (1 hour p.i.) are beneficial and if SPECT/CT acquisition should be preferred in H-mode (180° detector angle) or L-mode (90°).

**Methods:**

In SPECT/CT phantom measurements (NaI cameras, N = 2; CZT, N = 1), peak contrast recovery (CRpeak) was derived from sphere inserts or myocardial insert (cardiac phantom; signal-to-background ratio [SBR], 10:1 or 5:1). In 25 positive and 38 negative patients (reference: endomyocardial biopsy or clinical diagnosis), Perugini scores and heart-to-contralateral (H/CL) count ratios were derived from planar images 1 hour and 3 hour p.i.

**Results:**

In phantom measurements, accuracy of myocardial CRpeak at SBR 10:1 (H-mode, 0.95-0.99) and reproducibility at 5:1 (H-mode, 1.02-1.14) was comparable for H-mode and L-mode. However, L-mode showed higher variability of background counts and sphere CRpeak throughout the field of view than H-mode. In patients, sensitivity/specificity were ≥ 95% for H/CL ratios at both time points and visual scoring 3 hour. At 1 hour, visual scores showed specificity of 89% and reduced reader’s confidence.

**Conclusions:**

Early DPD images provided no additional value for visual scoring or H/CL ratios. In SPECT/CT, H-mode is preferred over L-mode, especially if quantification is applied apart from the myocardium.

**Supplementary Information:**

The online version contains supplementary material available at 10.1007/s12350-021-02715-6.

## Resumen

**Antecedentes:**

En la gammagrafía con [ 99mTc]-DPD para amiloidosis cardiaca ATTR, se recomienda la adquisición de imágenes planares a las 3h p.i y SPECT/CT en modo L. Este estudio investigó si las imágenes planares más tempranas (1h p.i.) tienen beneficios y si la adquisición del SPECT/CT debería preferirse en modo H (ángulo de detección de 180 °) o en modo L (90 °).

**Métodos:**

En las mediciones con el fantoma del SPECT/CT (cámaras de NaI, n= 2; CZT, n=1), la recuperación pico de contraste (CRpico) se derivó de inserciones de esfera o de inserciones miocárdicas (fantoma cardiaco; relación señal-fondo [SBR], 10:1 o 5: 1). En 25 pacientes positivos y 38 negativos (referencia: biopsia endomiocárdica o diagnóstico clínico), el score de Perugini y la relación de cuentas corazón-contralateral (H/CL) se derivaron de imágenes planares a 1h y 3h p.i.

**Resultados:**

En mediciones con el fantoma, la precisión del CRpico miocárdico en SBR 10:1 (modo H, 0.95-0.99) y la reproducibilidad a 5:1 (modo H, 1.02-1.14) fue comparable para el modo H y el modo L. Sin embargo, el modo L mostró una mayor variabilidad de las cuentas de fondo y del CRpico de la esfera en todo el campo de visión que el modo H. En pacientes, la sensibilidad/especificidad fue ≥ 95% para las relaciones H/CL en ambos puntos de tiempo y con un score visual a las 3 h. A 1h, los scores visuales mostraron una especificidad de (89%) y disminución de la confianza del lector.

**Conclusiones:**

Las imágenes tempranas de DPD no proporcionaron ningún valor adicional para el score visual o las relaciones H/CL. En SPECT/CT, se prefiere el modo H sobre el modo L, especialmente si la cuantificación se aplica aparte del miocardio.

## 摘要

**背景:**

[99mTc]Tc-DPD闪烁显像检查心肌ATTR淀粉样变性时, 建议使用3h p.i.平面图像和L模式SPECT/CT扫描。本研究探讨了早期平面图像(1h p.i.)是否有益, 以及SPECT/CT采集是否应首选H模式(探测器角度180°)或L模式(90°)。

**方法:**

在SPECT/CT模体测量中(NaI相机, n=2;CZT, n=1), 峰值造影剂恢复(CRpeak)来自球体插入物或心肌插入物(心脏模体; 信号与背景比[SBR], 10:1 或5:1)。在25个阳性和38个阴性病人中(参考标准: 心内膜活检或临床诊断), Perugini分数和心脏/对侧 (H / CL)计数比率来自平面图像1 h和3 h p.i.。

**结果:**

在模体测量中, 心肌CRpeak的SBR准确性 10:1 (H-mode, 0.95 - 0.99)和可重复性5:1 (H-mode, 1.02 - 1.14), H模式和L 模式相当。然而, 与H模式相比, L模式在整个视野中表现出更高的背景计数和球形CRpeak变异性。在患者中, 两个时间点的H/CL比值以及3h视觉评分的敏感性/特异性均≥95%。1h时, 视觉评分特异性为89%, 阅读者的信心降低。

**结论:**

DPD早期图像对视觉评分和H/CL比值没有额外的价值。在SPECT/CT中, H模式优于L模式, 特别是心肌之外定量时。

## Background

Amyloid transthyretin (ATTR) amyloidosis is a potentially life-threatening cause of heart failure caused by accumulation of liver-derived, misfolded transthyretin. Scintigraphy with bisphosphonates, such as [^99m^Tc]Tc-3,3-diphosphono-1,2-propanodicarboxylic acid (DPD), plays a key role in identifying myocardial involvement.^1^ Quantification of myocardial uptake using single photon emission computed tomography/computed tomography (SPECT/CT) might provide prognostic value^2^ or therapy monitoring of the multitude of recently introduced ATTR amyloidosis-specific drugs.^3^-^5^

A recent multi-institutional consensus report defined a standardized method of imaging.^6^,^7^ It was advocated to perform planar images with [^99m^Tc]Tc-DPD at (2-)3 hour p.i. In contrast, an earlier time point at 1 hour p.i. is recommended if [^99m^Tc]Tc-pyrophosphate (PYP) is used, while additional imaging at 3 hour p.i. would be recommended if cardiac uptake is superimposed by high blood pool activity at 1 hour p.i.^6^ Consensus has been reached to use the visual grading system proposed by Perugini^8^ and to derive semi-quantitative heart-to-contralateral (H/CL) count ratios from planar images.^6^ Furthermore, SPECT/CT in a dedicated cardiac acquisition protocol using L-mode (90°) detector configuration is recommended, while H-mode acquisition (i.e., parallel detector position) would only be optional.^7^ However, it has not yet been demonstrated if either acquisition mode provides superior quantitative accuracy.

Castano et al. reported higher diagnostic accuracy for cardiac involvement using [^99m^Tc]Tc-PYP in patients examined at 1 hour p.i. compared to patients examined at 3 hour p.i. (visual scoring and H/CL ratios).^9^ However, potential benefits of 1 hour vs 3 hour p.i. planar images have not been examined for [^99m^Tc]Tc-DPD.

The current investigation aimed at determining if—in line with [^99m^Tc]Tc-PYP—early planar images (1 hour p.i.) provide additional diagnostic value using [^99m^Tc]Tc-DPD. Furthermore, extensive comparative phantom measurements were performed to assess if H- or L-mode acquisition provides superior accuracy and reproducibility of myocardial DPD quantification. Three different dual-head general-purpose SPECT/CT cameras were compared, equipped with either sodium iodide (NaI) or cadmium zinc telluride (CZT) detectors.

## Methods

### SPECT/CT Phantom Measurements: Phantom Filling

Methods regarding the phantom measurements are described in Online Resource 1. Imaging was performed with three general-purpose SPECT/CT cameras. The NaI cameras were equipped with a low-energy high-resolution (LEHR) collimator (GE Discovery 670 DR Pro, GE Healthcare, Milwaukee, WI, USA; Siemens Symbia T6, Siemens Healthcare, Erlangen, Germany). The CZT camera (GE Discovery 670 CZT) used a wide-energy high-resolution (WEHR45) collimator.

### Patients: Characteristics

Between 05/2019 and 08/2020, 69 patients were referred consecutively for [^99m^Tc]Tc-DPD scintigraphy for suspicion of cardiac ATTR amyloidosis. Suspicion was either based on (a) biopsy-proven ATTR amyloidosis in other organs, (b) typical findings of cardiac amyloidosis in echocardiography (left ventricular wall thickness > 12 mm with diastolic dysfunction and “apical sparing” in longitudinal strain assessment) or magnetic resonance imaging (increased extracellular volume > 0.4 and positive late gadolinium enhancement) or (c) heart failure with preserved left ventricular ejection fraction of unclear etiology. In 63 of these 69 patients (45 men), a standard of reference (SOR) was available, and these patients were included into this retrospective analysis. Positive SOR (25 of 63 patients) required histological diagnosis of cardiac ATTR amyloidosis by endomyocardial biopsy (N = 21) or by extracardiac biopsy combined with typical findings in echocardiography or cardiac MRI (N = 4) according to consensus recommendations.^10^ Negative SOR (38 of 63 patients) required either endomyocardial biopsy negative for amyloidosis (N = 8) or positive for AL amyloidosis (N = 5) or negative clinical criteria (i.e., absence of typical findings of echocardiography and MRI with [N = 4] or without [N = 21] extracardiac proof of ATTR amyloidosis). Median age was 77 years (interquartile range [IQR] 69 to 81 years; range 34 to 88 years). All procedures were in accordance with the ethical standards of the Charité ethics commission, and all patients gave their informed consent to participate.

### Patients: Acquisition of Planar Images

A median of 13.5 mCi (500 MBq) [^99m^Tc]Tc-DPD (IQR 13.1 to 14 mCi [483 to 518 MBq]) corresponding to 0.18 mCi·kg (6.6 MBq·kg; IQR, 0.16 to 0.21 mCi·kg [6.0 to 7.7 MBq·kg]) was injected intravenously. Using one of the three dual-head cameras that were used for the phantom measurements, a static planar image (anterior and posterior view) of the thorax was obtained after a median of 64 minutes (IQR 58 to 73 minutes) p.i. and at 207 minutes p.i. (IQR 197 to 225 minutes). Images were acquired for 5 min each (matrix size, 256 × 256).

SPECT/CT data from patients were not analyzed because the true activity concentration in the myocardium is not known.

### Patients: Heart-to-Contralateral (H/CL) Ratio in Planar Images

A circular region of interest (ROI) was placed in the left hemithorax covering the heart and adjacent ribs. This ROI was mirrored to the right hemithorax to calculate the H/CL ratio by dividing the mean counts per pixel in the left and the right hemithorax ROI.

### Patients: Visual Score

Visual scoring was performed independently by two experienced nuclear medicine physicians using the planar thorax images at 1 hour and 3 hour p.i. (reader 1, > 10 years of experience; reader 2, > 5 years). Reading of the 1 hour time point was performed first and blinded to the 3 hour image. Both readers were blinded from any clinical information of the patients. In addition to independent reading, a consensus score was determined in discordant cases. Score 0 was defined as absence of any specific cardiac uptake, score 1 was cardiac uptake < rib uptake, score 2 was cardiac uptake ≥ ribs, and score 3 was cardiac uptake markedly > rib uptake (reduced bone uptake was optional).^8^

In addition, each reader rated the confidence in assigning the visual score at each time point with a 4-point scale (1, highly uncertain; 2, rather uncertain; 3, rather certain; 4, highly certain).

### Statistical Analysis

Statistical analysis was performed using SPSS 22 (IBM Corporation, Armonk, NY, USA). In phantom data, paired *t*-test was used to compare volume sensitivities or relative errors between cameras. Peak contrast recovery (CRpeak) values from phantom measurements were compared between H-mode and L-mode using a one-sample *t*-test (only a single measurement with L-mode; Table 1).

**Table 1:** Sphere CRpeak differences between H-mode and L-mode

Camera	Sphere (mm)	H-mode	L-mode	*P* value
DR Pro	37	1.00 ± 0.007	0.94	**< 0.01**
	28	1.05 ± 0.01	0.95	**< 0.01**
	22	0.87 ± 0.03	0.58	**< 0.01**
	17	0.59 ± 0.04	0.34	**< 0.01**
	13	0.32 ± 0.03	0.23	**< 0.05**
	10	0.19 ± 0.02	0.13	**< 0.05**
CZT	37	1.00 ± 0.03	1.12	**< 0.05**
	28	1.02 ± 0.004	1.04	**< 0.05**
	22	0.84 ± 0.01	0.58	**< 0.01**
	17	0.59 ± 0.06	0.3	**< 0.05**
	13	0.36 ± 0.02	0.19	**< 0.01**
	10	0.23 ± 0.01	0.15	**< 0.01**
Symbia	37	1.14 ± 0.04	1.5	**< 0.01**
	28	1.05 ± 0.03	0.99	0.074
	22	0.63 ± 0.0	0.46	**< 0.001**
	17	0.4 ± 0.03	0.19	**< 0.01**
	13	0.22 ± 0.01	0.14	**< 0.01**
	10	0.16 ± 0.01	0.12	**< 0.05**

Sphere CRpeak were derived with H-mode (serial examination with 3 scans) or L-mode (single examination). Results of the one-sample *t*-test are provided. L-mode generally showed higher deviations from the optimum (CR = 1.0).

H/CL ratios at 1 hour and 3 hour p.i. were compared with the Wilcoxon signed-rank test. Agreement between visual scores (0 to 3) at 1 hour and 3 hour p.i. or between both readers was rated by intraclass correlation ICC(A,1) and its 95%-confidence interval (95% CI) according to^11^ (two-way mixed model, single measurement, absolute agreement) and interpreted according to.^12^ Receiver operating characteristic curves and areas under the curve (AUC) were derived from H/CL ratios based on the SOR. Using Youden’s index, optimal cut-offs were determined (H/CL ratio ≥ 1.6 at both time points), and resulting sensitivity and specificity were compared between both time points using McNemar’s test (two-sided). The paired t-test compared confidence of the visual scoring between both time points. Statistical significance was assumed at *P* < 0.05.

## Results

### Phantom Measurements: Comparison of H-Mode and L-Mode

#### Myocardial CRpeak

At signal-to-background ratio (SBR) of 10:1, CRpeak of the myocardial compartment was similar between H-mode (0.99 ± 0.07) vs L-mode (1.00; *single measurement*, *P* = 0.84) for the DR Pro camera, for the CZT camera (0.95 ± 0.05 vs 1.01, *P* = 0.17) and for the Symbia camera (0.96 ± 0.08 vs 1.01 ± 0.02, *P* = 0.35).

At SBR of 5:1 (DR Pro and CZT camera only), the DR Pro and CZT showed similar myocardial CRpeak for H-mode vs L-mode (DR Pro, 1.09 ± 0.1 vs 1.11, *P* = 0.76; CZT, 1.14 ± 0.13 vs 1.15, *P* = 0.96).

#### Sphere CRpeak and CRmax

**Figure 1:**
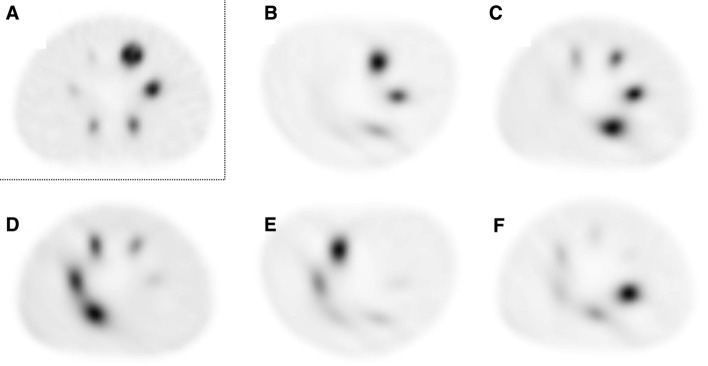
NEMA IEC phantom positioning for H-mode vs L-mode. Transaxial SPECT slices depicting the six sphere inserts of the NEMA IEC phantom obtained with H-mode with automated body contouring (**A**) or in serial L-mode acquisitions with varying positioning of the phantom **(B-F**) (always Symbia camera). Only with H-mode acquisition are all six spheres visually detectable and appear roughly spherical. In contrast, spheres are blurred, deformed, and less contrasted with L-mode. This is especially apparent for the spheres located in the right posterior segment (i.e., in furthest distance from the detectors) (**D-F**)

**Figure 2:**
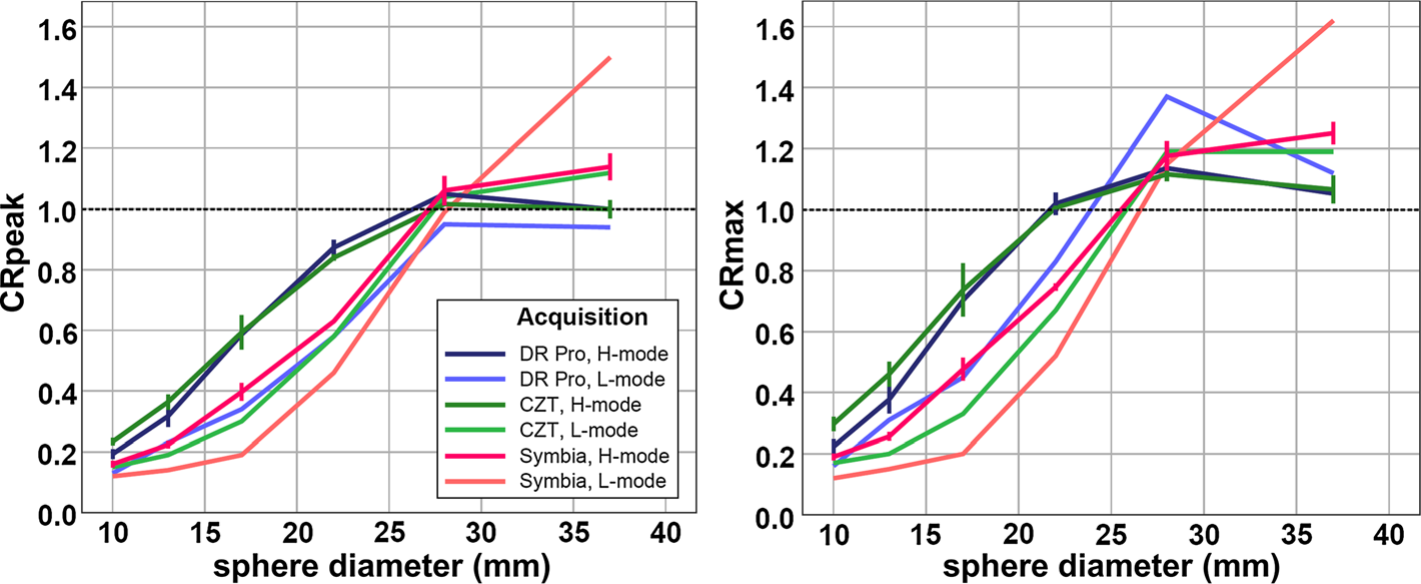
NEMA IEC phantom spheres: CRpeak and CRmax. CRpeak and CRmax of the NEMA IEC phantom spheres with all three cameras separated by H-mode and L-mode. In H-mode acquisitions, the lines represent the mean CR of three serial measurements after one initial filling (error bars: ±1 SD); L-mode acquisition was a single measurement. The dashed lines provide the optimal CR of 1.0 as reference. CR with H-mode are closer to 1.0 than with L-mode. CRpeak for the two largest spheres (diameter, 37 and 28 mm) are generally closer to 1.0 than corresponding CRmax

For each of the three cameras, H-mode provided more accurate CRpeak than L-mode for every sphere size (Table 1). CRmax showed higher overestimation of the true sphere activity concentration than CRpeak in the two largest spheres for all three cameras in both acquisition modes (Figures 1, 2).

#### Variability in NEMA IEC phantom background counts

The coefficient of variation of NEMA IEC phantom background counts was lower for H-mode vs L-mode for the DR Pro (4.0% ± 0.5% vs 7.5% ± 2.3%, *P* = 0.07), the CZT (1.1% ± 0.3% vs 3.4% ± 1.0%, *P* = 0.03), and the Symbia (2.9% ± 0.1% vs 4.6% ± 0.7%, *P* = 0.01).

#### L-mode: variability of sphere CRpeak

Using L-mode acquisition with the Symbia, CRpeak for each of the six spheres varied substantially depending on their localization in the transaxial field of view (Table 2).

**Table 2:** Variation of sphere CRpeak in L-mode

Camera	Sphere (mm)	L-mode	H-mode (different measurement)
Symbia	37	0.99 to 1.62	1.14 ± 0.04
	28	0.59 to 1.2	1.05 ± 0.03
	22	0.31 to 0.88	0.63 ± 0.0
	17	0.2 to 0.42	0.4 ± 0.03
	13	0.15 to 0.21	0.22 ± 0.01
	10	0.1 to 0.17	0.16 ± 0.01

This table provides the range of CRpeak observed with L-mode depending on the variation in sphere localization, which resulted from repeated acquisitions of the NEMA IEC phantom after rotating the phantom or turning it from supine to prone position (Symbia camera only). For comparison, mean ± SD of CRpeak with H-mode are provided, which were obtained from serial measurements without changing the phantom position (see also Table 1).

CRpeak of all spheres was on average lower with L-mode than with H-mode in the right posterior segment of the field of view (mean relative difference, − 29.9 ± 22.3%), in the right anterior (− 7.6 ± 21.0%) and left posterior segment (− 7.6 ± 20.3%; Table 3). In contrast, average CRpeak were higher with L-mode than H-mode in the left anterior segment (+ 9.1 ± 24.9%).

**Table 3:** CRpeak depending on sphere localization in L-mode

Right anterior		Left anterior	
37 mm 28 mm 22 mm 17 mm 13 mm 10 mm Mean ± SD	+ 29% − 25% + 5% − 11% − 18% − 25% − 7.6 ± 21.0%	37 mm 28 mm 22 mm 17 mm 13 mm 10 mm Mean ± SD	+ 42% + 14% + 30% + 5% − 18% − 19% + 9.1 ± 24.9%
Right posterior		Left posterior	
37 mm 28 mm 22 mm 17 mm 13 mm 10 mm Mean ± SD	− 13% − 41% − 49% − 50% − 32% + 6% − 29.9 ± 22.3%	37 mm 28 mm 22 mm 17 mm 13 mm 10 mm Mean ± SD	+ 24% + 12% − 17% − 23% − 23% − 19% − 7.6 ± 20.3%

Relative differences in CRpeak between L-mode and H-mode are given for each of the four segments of the transaxial field of view (Symbia only). L-mode underestimated CRpeak compared to H-mode in both right segments and the left posterior segment but overestimated CRpeak in the left anterior segment (=heart). The latter is closest to the detectors in L-mode.

### Patient Examinations (Planar Images Only)

The SOR was positive for cardiac ATTR amyloidosis in 25 of 63 patients (40%) and negative in 38 patients (60%; Table 4). Among the 25 patients with positive SOR, 24 patients were DPD positive based on the planar images (i.e., visual consensus score ≥ 2 at 3 hour p.i.) resulting in a sensitivity of 96% (95% CI 80 to 100%; Table 4). Conversely, 36 of 38 patients with negative SOR were DPD negative (specificity, 95%; 95% CI 82 to 99%).

**Table 4:** Diagnostic accuracy of planar imaging at 1 hour and 3 hour p.i.

Patient groups	1 hour p.i. correct		3 hour p.i. correct		*P* value
	Visual score	H/CL ratio	Visual score	H/CL ratio	(1 hour vs 3 hour)
Positive SOR (N = 25)					
(1) Biopsy-proven cardiac ATTR amyloidosis	21/21	20/21	20/21	20/21	
(2) Proven systemic ATTR amyloidosis, and echo and/or MRI typical of cardiac amyloidosis	4/4	4/4	4/4	4/4	
Combined sensitivity (95% CI)	100 (86 to 100)%	96 (80 to 100)%	96 (80 to 100)%	96 (80 to 100)%	1.0/1.0
Negative SOR (N = 38)					
(1) Biopsy-excluded cardiac ATTR amyloidosis	8/8	8/8	8/8	8/8	
(2) Biopsy-proven cardiac AL amyloidosis	2/5	4/5	4/5	4/5	
(3) Echo and/or MRI not typical of amyloidosis, and no proof of extracardiac ATTR amyloidosis	21/21	21/21	21/21	21/21	
(4) Proven extracardiac ATTR amyloidosis, but echo and/or MRI not typical of cardiac involvement	3/4	4/4	3/4	3/4	
Combined specificity (95% CI)	89 (75 to 97)%	97 (86 to 100)%	95 (82 to 99)%	95 (82 to 99)%	0.5/1.0

True positive, true negative cases, sensitivity and specificity (with 95% confidence intervals [95% CI]) are displayed for visual scores (positive, ≥ 2) and H/CL ratios (positive, ≥ 1.6). Echocardiography typical of cardiac amyloidosis included left ventricular wall thickness >12 mm, diastolic dysfunction and “apical sparing” in longitudinal strain. Typical MRI included increased extracellular volume > 0.4 and late gadolinium enhancement. *P* values (McNemar’s test) are provided for visual scores/H/CL ratios comparing 1 hour vs 3 hour p.i.

#### Visual assessment: 1 hour vs 3 hour p.i.

Interrater agreement was excellent at both time points but slightly lower at 1 hour p.i. (ICC 0.95; 95% CI 0.91 to 0.97) than at 3 hour p.i. (ICC 1.0; 95% CI 0.99 to 1.0).

A contingency table of visual consensus scores at 1 hour and 3 hour p.i. is provided in Table 5. Agreement of visual consensus scores at 1 hour vs 3 hour p.i. was excellent (ICC 0.85; 95% CI 0.39 to 0.94). Visual consensus scores at 1 hour vs 3 hour p.i. resulted in a comparable sensitivity of 100% (95% CI 86 to 100%) vs 96% (95% CI 80 to 100%; McNemar’s test, *P* = 1.0). Specificity was slightly lower at 1 hour (89%; 95% CI 75 to 97%) than at 3 hour p.i. (95%; 95% CI 82 to 99%; *P* = 0.5).

**Table 5:** Comparison of visual consensus scores at 1 hour and 3 hour p.i.

3 hour 1 hour	0	1	2	3	Total
0	8	0	0	0	8
1	26	0	0	0	26
2	1	2	0	0	3
3	0	0	2	24	26
Total	35	2	2	24	63

Contingency table for visual scores (reader consensus) for planar images at 1 hour and 3 hour p.i.

#### Confidence of the visual assessment

**Figure 3:**
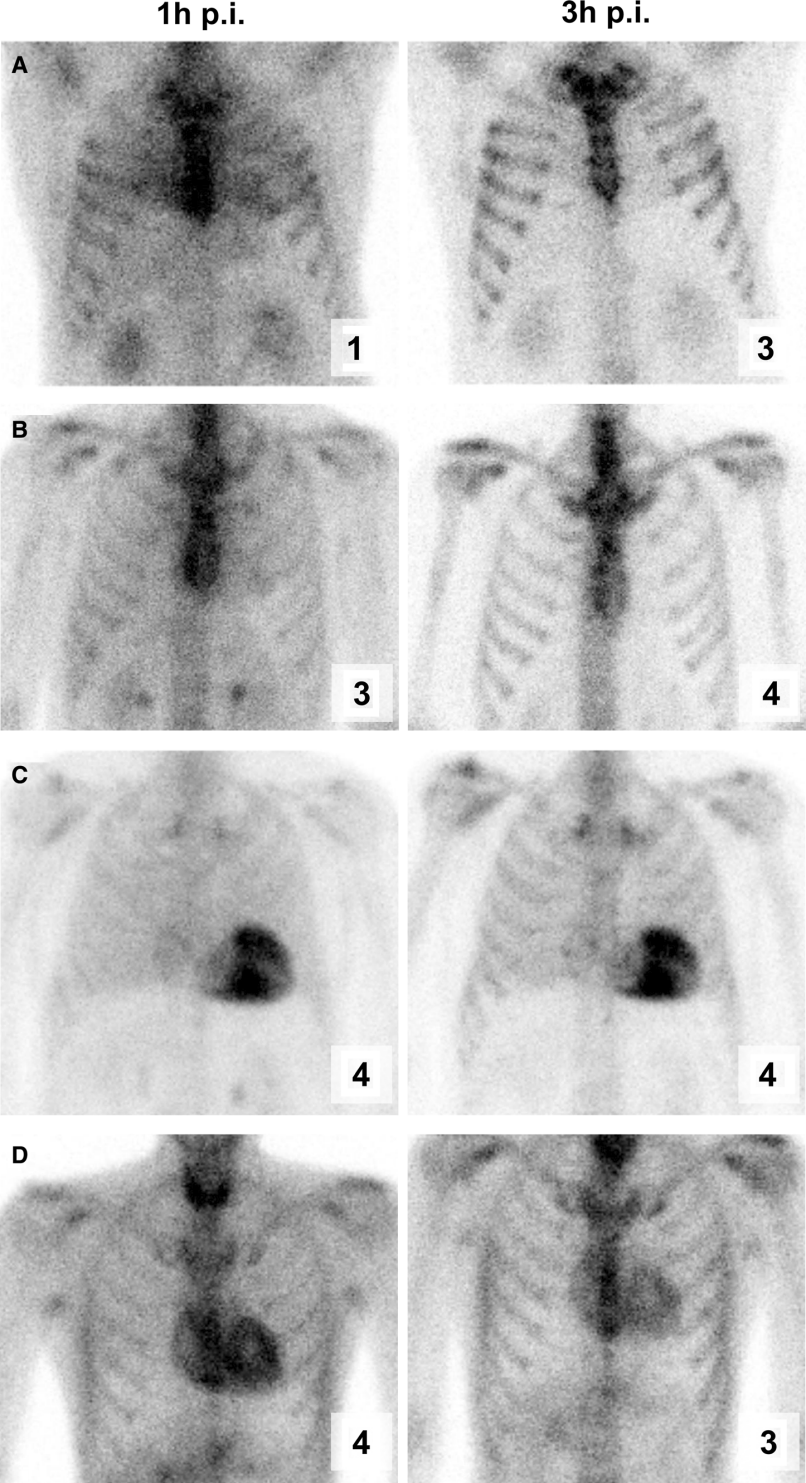
Confidence during visual assessment. Patient examples showing the confidence score (right lower corner) that rated the reader’s confidence in assigning a specific visual score 0-3 (note: not the confidence for the binary decision of a positive vs negative case). Each image is scaled to its individual count maximum. **A** 53-year-old male with systemic ATTR amyloidosis but cardiac involvement excluded by biopsy. **B** 42-year-old male with systemic ATTR amyloidosis but echocardiography and clinical status not suggestive of cardiac involvement. **C** 77-year-old male with biopsy-proven cardiac ATTR amyloidosis. **D** 39-year-old male with proven systemic hereditary ATTR amyloidosis but repeated echocardiography over 3 years not suggestive of cardiac involvement

The mean confidence of both readers in assigning a specific visual score was significantly lower at 1 hour (reader 1, 2.9 ± 1.0; reader 2, 3.4 ± 0.8) compared to 3 hour p.i. (reader 1, 3.6 ± 0.6; reader 2, 3.8 ± 0.5; each *t*-test, *P* < 0.001; Figure 3).

#### H/CL ratio: 1 hour vs 3 hour p.i.

**Figure 4:**
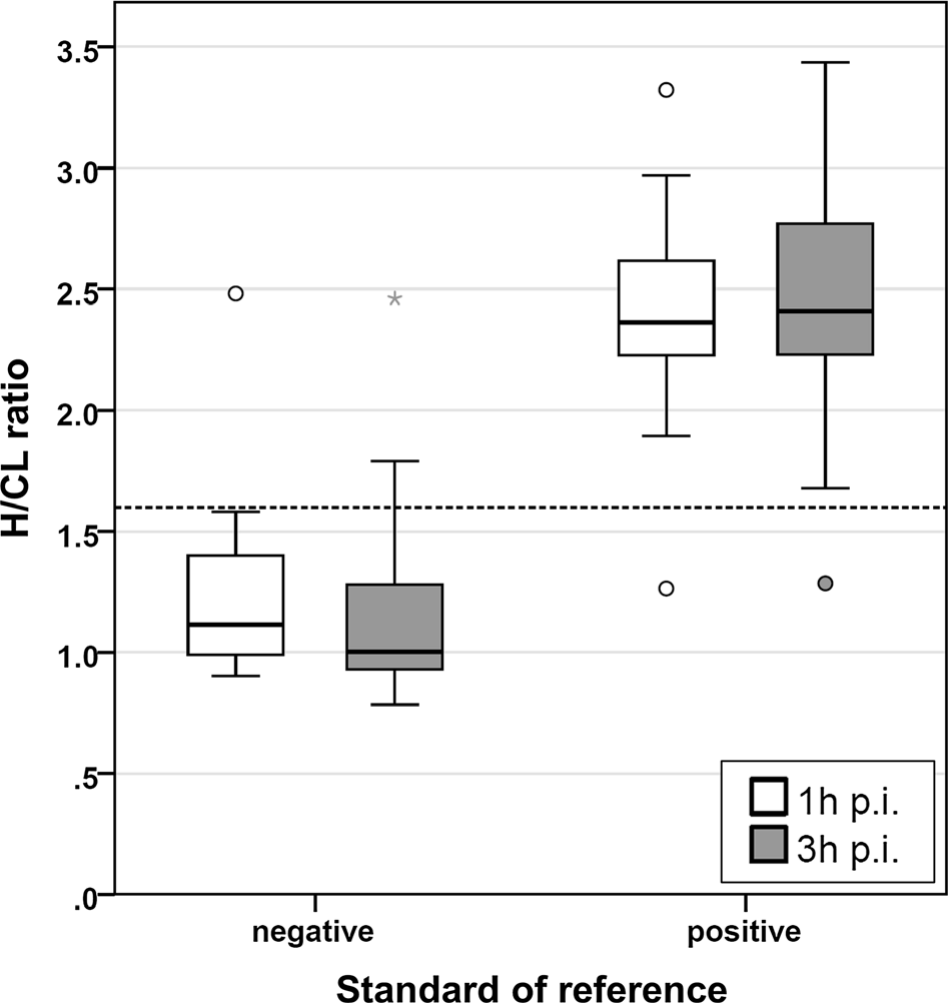
Box plots of H/CL ratios at 1 hour and 3 hour p.i. Box plots of H/CL ratios at 1 hour and 3 hour p.i. Patients are separated according to the standard of reference. Circles and asterisks highlight extreme values and outliers. The negative case with H/CL ratios of approximately 2.5 was a patient with cardiac AL amyloidosis. The dashed horizontal line represents the optimal cut-off of 1.6 for both time points

In patients with a negative SOR, the median H/CL ratio at 1 hour p.i. was 1.1 (IQR 1.0 to 1.4; range 0.9 to 2.5), which was slightly but significantly higher than at 3 hour p.i. (median 1.0; IQR 0.9 to 1.2; range 0.8 to 2.5; *P* < 0.001; Figure 4). In contrast, in patients with a positive SOR, median H/CL ratio at 1 hour p.i. was 2.4 (IQR 2.2 to 2.6; range 1.3 to 3.3) and similar to 3 hour p.i. (median 2.4; IQR 2.2 to 2.8; range 1.3 to 3.4; *P* = 0.25).

**Figure 5:**
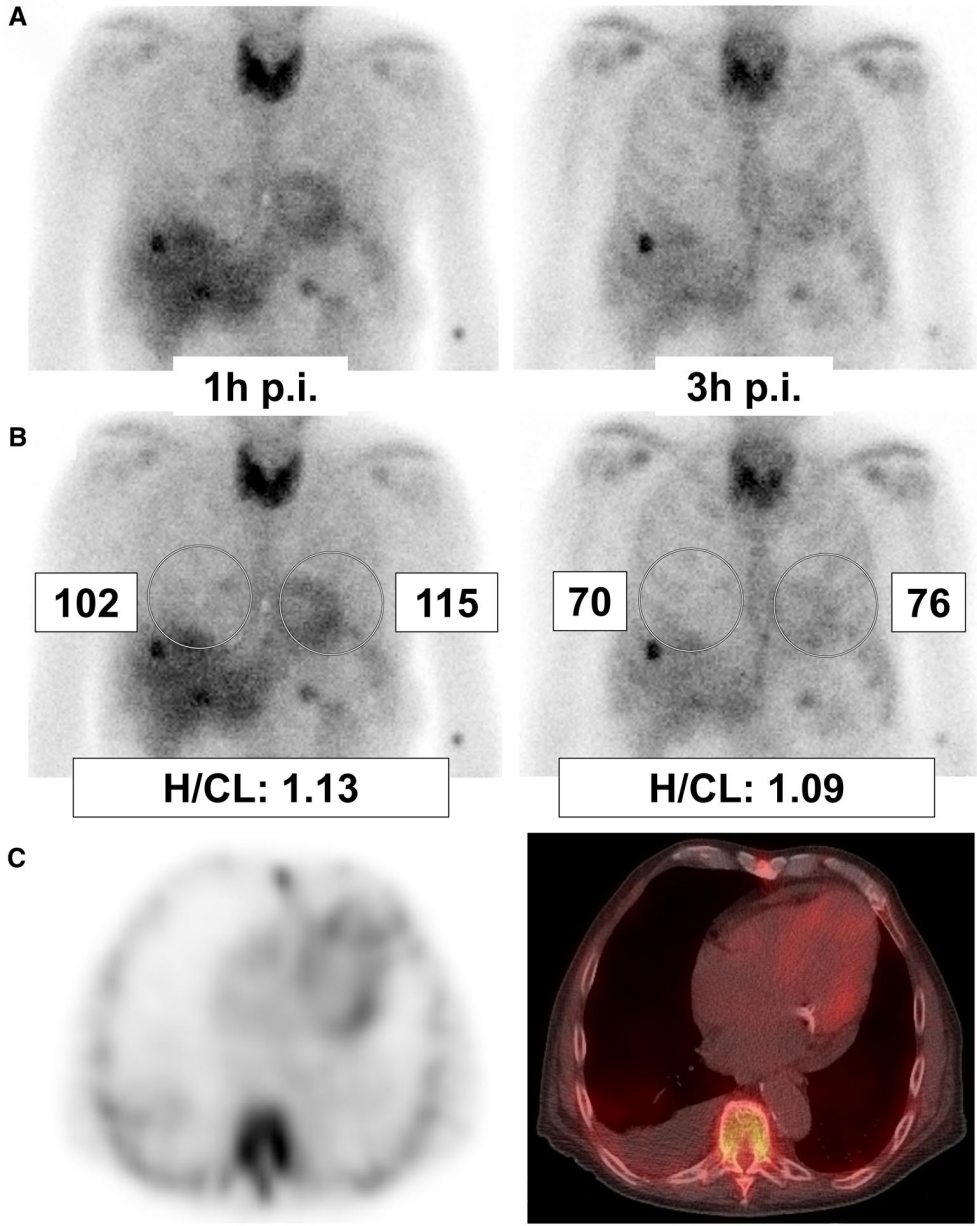
Patient example. 79-year-old male with proven AL amyloidosis. Planar anterior images at 1 hour and 3 hour p.i. are displayed (**A** raw images; **B** with regions of interest [ROIs], mean counts and H/CL ratios). Both time points show pathological uptake in the thyroid gland and liver due to AL amyloidosis. At 1 hour p.i. (**A** left), blood pool-related activity in the heart led to a visual score of 2 (i.e., false positive). At 3 hour p.i. (**A** right), visual assessment was impaired by hepatic uptake; the visual score was 1 (true negative). SPECT and SPECT/CT (**C**) confirmed mild myocardial uptake, which can be observed in AL amyloidosis. In contrast to visual assessment, H/CL ratios were unequivocally true negative at both time points, and despite the different visual appearance of the heart, H/CL ratio was only marginally higher at 1 hour than at 3 hour p.i. (**B**). This may be explained by the simultaneous increase of counts in the right hemithorax ROI resulting from increased blood pool at 1 hour p.i., which is disregarded during visual assessment

Corresponding AUC for H/CL ratios at 1 hour or 3 hour p.i. were 0.97 (95% CI 0.93 to 1.0) or 0.98 (95% CI 0.94 to 1.0), respectively. The optimal cut-off was ≥ 1.6 for both time points. This resulted in a sensitivity and specificity at 1 hour p.i. of 96% (95% CI 80 to 100%) and 97% (86 to 100%; Table 4; Figure 5), which was comparable to 3 hour p.i. with a sensitivity and specificity of 96% (80 to 100%) and 95% (82 to 99%; each *P* = 1.0).

If the previously proposed cut-off of ≥ 1.5^9^,^13^ was used, both time points showed identical sensitivity and specificity of 96% (80 to 100%) or 92% (79 to 98%), respectively (each *P* = 1.0).

## Discussion

This study examined a comprehensive imaging protocol for [^99m^Tc]Tc-DPD scintigraphy in patients with suspicion for cardiac ATTR amyloidosis.

Applying the visual score for planar images initially proposed by Perugini et al.,^8^ sensitivity at the early time point at 1 hour p.i. (100%) was comparable to 3 hour p.i. (96%). The slightly lower specificity at the early time point (89% vs 95%) might be caused by high blood pool activity of DPD at 1 hour p.i. This is underlined by the observation that specificity at 1 hour p.i. was not reduced if H/CL ratios were used. High blood pool activity unspecifically increases counts in both hemithorax ROIs. In the right hemithorax, counts are increased by pulmonary and soft tissue perfusion while in the left hemithorax, the intraventricular blood pool additionally increases counts. Due to this relatively balanced increase on both sides, H/CL ratios remained < 1.5 in DPD-negative patients, and specificity was unaffected (Figure 5). In contrast, isolated inspection of the heart vs ribs for visual assessment may overestimate myocardial uptake and reduce specificity. This also led to lower confidence in visual reading at 1 hour than at 3 hour p.i.

It may be noted that specificity of visual assessment at both time points may have been higher than given in Table 4. A single patient who was classified as “negative SOR” because findings in echocardiography and MRI were non-typical of cardiac amyloidosis may have been false negative by these modalities. The patient had extracardiac proof of ATTR amyloidosis. Due to negative echocardiography and MRI, criteria for diagnosis of cardiac amyloidosis were not fulfilled according to current consensus 10. DPD was positive according to visual scores of 3 at both time points (both H/CL ratios were < 1.5). According to previous reports,^14^,^15^ scintigraphy can be positive in cases of early cardiac ATTR involvement which is not detected by echocardiography. MRI with late gadolinium sequences may also be false negative for cardiac amyloidosis (sensitivity of 85%).^16^ Therefore, diagnosis of cardiac amyloidosis would currently require a positive endomyocardial biopsy or amyloid-specific positron emission tomography (PET); however, both were not performed as it was decided that there would be no clinical benefit in this individual case.

In general, H/CL ratios might be falsely low due to reduced DPD uptake in the area of a previous myocardial infarction. In the current analysis, 10 patients had documented previous myocardial infarction. In 6 of these 10 patients, H/CL ratios at either time point were negative. SPECT/CT images in these cases confirmed that DPD uptake was absent in the whole left ventricular myocardium (i.e., not only focally absent).

To the best of our knowledge, no other reports on early vs late planar images for [^99m^Tc]Tc-DPD scintigraphy are available. However, the current results partly differ from Castano *et al.* who used [^99m^Tc]Tc-PYP in 171 patients with suspicion for cardiac ATTR amyloidosis. Similar to the current analysis, visual assessment in 126 patients examined at 1 hour p.i. showed specificity of 79%, which was lower than 100% in 45 different patients scanned at 3 hour p.i. However, in contrast to the current results, sensitivity in patients examined at 3 hour p.i. was substantially lower than in patients with early imaging (58% vs 95%). Notably, intra-individual comparison was not provided, and comparability of sensitivity and specificity between both groups could be limited. The discrepant sensitivity at 3 hour p.i. between both studies may not be explained by the different tracer used, because similar blood pool clearance has been reported for [^99m^Tc]Tc-DPD and [^99m^Tc]Tc-PYP in a rat model.^17^ Regarding H/CL ratios, Castano et al. reported slightly higher diagnostic accuracy at 1 hour than 3 hour p.i.^9^ while accuracies were comparable in the present analysis.

Based on the current patients, the early time point may at best achieve similar diagnostic accuracy as the late time point with [^99m^Tc]Tc-DPD (H/CL ratios) or even inferior accuracy and reader confidence (visual score). Therefore, in contrast to [^99m^Tc]Tc-PYP imaging, the standard time point for planar images with [^99m^Tc]Tc-DPD should remain at 3 hour p.i., which also allows for a concise imaging protocol (3 hour p.i. is recommended for SPECT or SPECT/CT^7^).

Another source of controversy relates to the optimal SPECT/CT acquisition protocol for quantification of myocardial uptake. The current consensus of several international societies of cardiology or nuclear medicine^7^ recommends L-mode detector configuration while H-mode acquisition would be optional. The current phantom measurements show that—in principle—optimized reconstruction protocols for each camera and each acquisition mode enable accurate and reproducible quantification of myocardial [^99m^Tc]Tc-DPD uptake with both H-mode and L-mode if the myocardial CRpeak is used. However, the current comparative phantom measurements demonstrate that quantitative accuracy with L-mode can differ substantially throughout the transaxial field of view (e.g., in the liver or muscles). Notably, quantification of [^99m^Tc]Tc-DPD in reference organs outside the left anterior hemithorax (paraspinal muscles, vertebrae, liver) was recently proposed by Scully *et al.* in patients with suspicion for cardiac ATTR amyloidosis. The authors used H-mode with automated body contouring.^18^ In L-mode acquisition, detector rotation only covers the left hemithorax directly, and therefore the system’s sensitivity is higher in these locations closer to the detectors as opposed to the right hemithorax. This observation was made although image reconstruction for all three cameras included CT-based attenuation correction and, more specifically, 3D resolution recovery.^19^,^20^ Furthermore, sphere CRpeak showed substantial localization-dependent deviations compared to H-mode (Tables 2, 3), and underestimation of the true activity concentration in smaller spheres was generally more pronounced with L-mode (Figure 2; Table 1). Therefore, L-mode would not provide the same accuracy in lesions/organs outside the cardiac compartment^18^ or in small targets (e.g., right ventricle myocardium).

Consequently, H-mode with body contouring should be preferred over L-mode for quantitative purposes. In additional serial measurements using H-mode (Online Resource 2), myocardial CRpeak varied—on average—between 0.95 and 0.99 at SBR 10:1 using the three cameras. Such inter-camera variability of approximately ± 5% and variability between scans (1-2 hour difference) of ± 10% would be acceptable for inter- or intraindividual comparison in clinical application. Variability at SBR of 7:1 remained similar (average CRpeak between 1.0 and 1.04). This implies that in patients with less intense uptake or if uptake varies considerably between two examinations over time (e.g., in the context of new ATTR amyloidosis-specific drugs^3^-^5^), real differences of > 10% might be detectable.

CRpeak was decisive of diagnostic accuracy and reconstruction protocol optimization. CRmax may be more common, but usually overestimate sphere activity concentrations and only served for comparison.^21^-^23^ In contrast, mean counts usually underestimate focal activity concentration.^24^-^26^ The peak counts give an average value, are less susceptible to image noise and different reconstruction,^23^,^27^ and show superior test-retest repeatability than CRmax 28. Furthermore, because the peak value is obtained from a standardized volume of interest, it is also less susceptible than the mean counts to variability in volume delineation between observers^29^,^30^ or delineation algorithms.^31^,^32^ Consequently, only CRpeak would allow 100% recovery of the myocardial activity concentration in SPECT/CT data of patients while obviating time-consuming and error-prone delineation of the exact myocardial volume (without knowing the actual distribution volume of [^99m^Tc]Tc-DPD). Scully et al. demonstrated almost perfect differentiation of DPD positive and negative cases based on the cardiac peak standardized uptake value (SUVpeak) in 100 patients (AUC 0.999).^18^

To ensure representativeness for clinical conditions, filling of the cardiac phantom was based on the [^99m^Tc]Tc-DPD blood pool activity concentration that can be expected in patient examinations^17^,^33^-^35^ and based on SBR derived from own patient samples of positive DPD scans. However, underestimation of myocardial activity in patients could still result from cardiac movement due to breathing and ventricular contractions, although gated data acquisition would—ideally—compensate for the latter.^36^,^37^ Consequently, even after optimization of acquisition and reconstruction protocols with the static cardiac phantom, quantitative accuracy in patients remains unclear (with both H-mode and L-mode).

## New Knowledge Gained

To ensure accurate and reproducible quantification of cardiac SPECT/CT in patients with ATTR amyloidosis, the proposed workflow of optimized image acquisition (H-mode) and image reconstruction (based on the myocardial CRpeak in a cardiac phantom) can be employed. This facilitates comparable quantitative accuracy for myocardial uptake between different general-purpose SPECT/CT cameras (NaI and CZT detectors). Early planar images may be safely omitted with [^99m^Tc]Tc-DPD for a convenient diagnostic workflow.

## Conclusions

Early planar images (1 hour p.i.) can be omitted for [^99m^Tc]Tc-DPD as they provided no additional value for Perugini scoring or H/CL ratios compared to the reference at 3 hour p.i. In SPECT/CT phantom measurements, both H-mode and L-mode acquisition accurately quantified myocardial [^99m^Tc]Tc-DPD uptake using the CRpeak. However, L-mode would impair quantitative accuracy in localizations other than the heart. These results suggest that H-mode acquisition with automated body contouring is preferable and imply that the current consensus recommendations may require reevaluation.

## Supplementary Information

Below is the link to the electronic supplementary material.

Supplementary file1 (M4A 9267 kb)

Supplementary file2 (PPTX 629 kb)

## Abbreviations

95% CI: 95%-confidence interval
AL: Amyloid light-chain
ATTR: Amyloid transthyretin
CR: Contrast recovery
CZT: Cadmium zinc telluride
DPD: 3,3-Diphosphono-1,2-propanodicarboxylic acid
H/CL ratio: Heart-to-contralateral count ratio
NaI: Sodium iodide
PYP: Pyrophosphate
ROI: Region of interest
SBR: Signal-to-background ratio
SOR: Standard of reference
