# Supplementary material for: An optimized imaging protocol for [99mTc]Tc-DPD scintigraphy and SPECT/CT quantification in cardiac transthyretin (ATTR) amyloidosis
Source: J Nucl Cardiol. 2021 Jul 30;28(6):2483–96. doi: 10.1007/s12350-021-02715-6 (PMC8709821; doi:10.1007/s12350-021-02715-6)
Supplement: Supplementary file 2 — Supplementary file2 (PPTX 629 kb) [file 12350_2021_2715_MOESM2_ESM.pptx]

## Slide 1
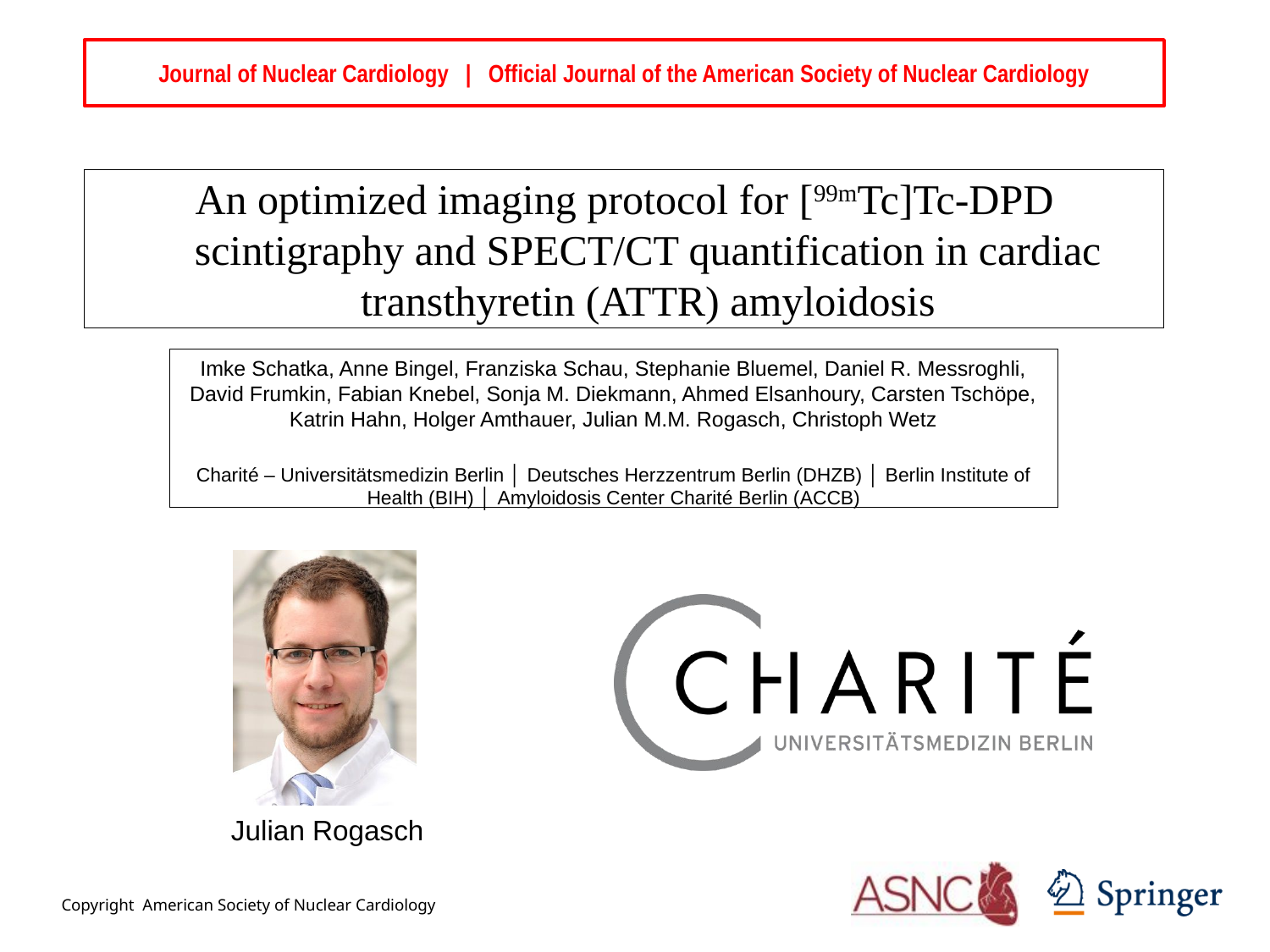

Journal of Nuclear Cardiology | Official Journal of the American Society of Nuclear Cardiology
# An optimized imaging protocol for [99mTc]Tc-DPD scintigraphy and SPECT/CT quantification in cardiac transthyretin (ATTR) amyloidosis
Imke Schatka, Anne Bingel, Franziska Schau, Stephanie Bluemel, Daniel R. Messroghli, David Frumkin, Fabian Knebel, Sonja M. Diekmann, Ahmed Elsanhoury, Carsten Tschöpe, Katrin Hahn, Holger Amthauer, Julian M.M. Rogasch, Christoph Wetz
Charité – Universitätsmedizin Berlin │ Deutsches Herzzentrum Berlin (DHZB) │ Berlin Institute of Health (BIH) │ Amyloidosis Center Charité Berlin (ACCB)
Julian Rogasch
Copyright American Society of Nuclear Cardiology

## Slide 2
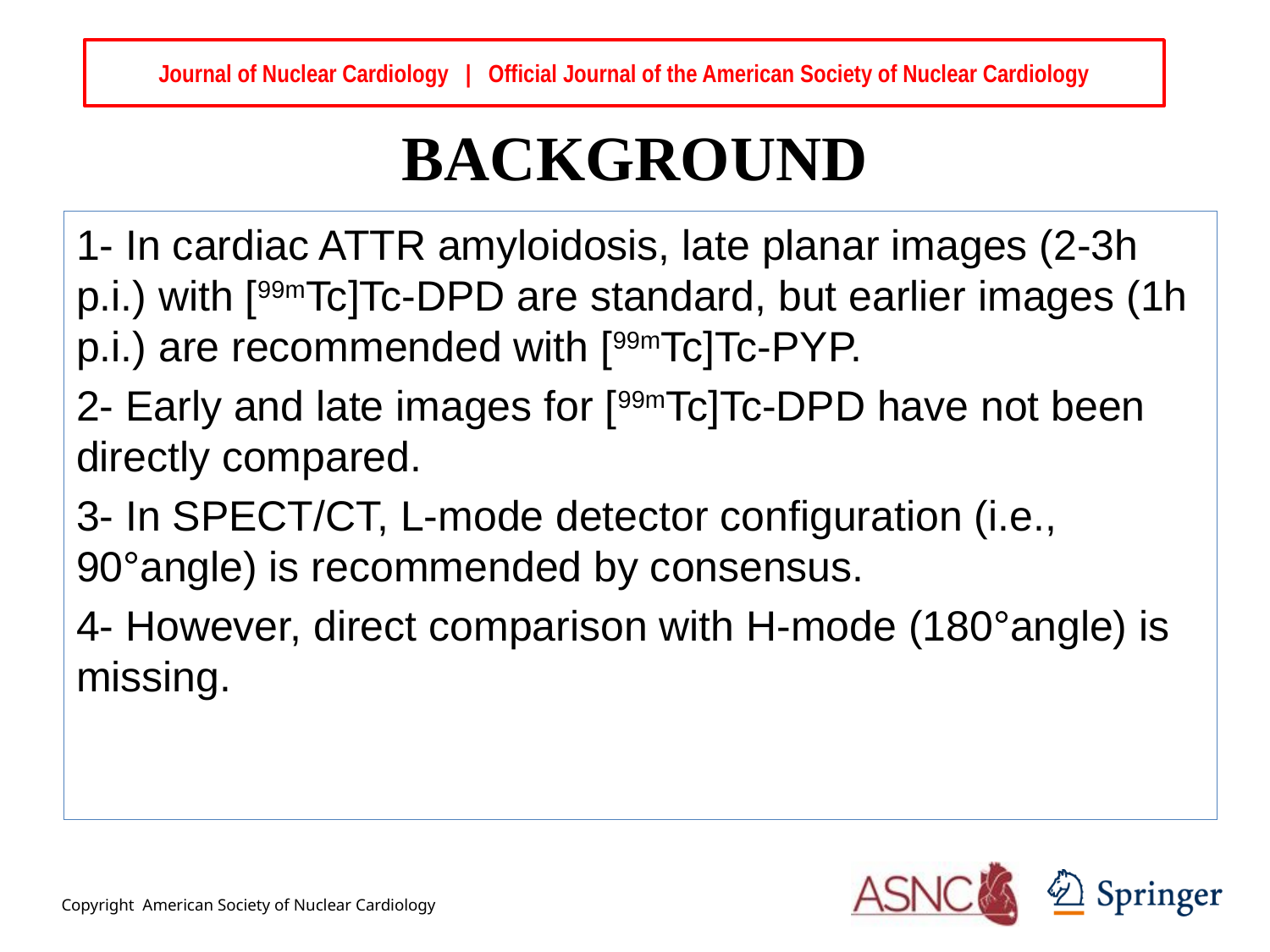

Journal of Nuclear Cardiology | Official Journal of the American Society of Nuclear Cardiology
# BACKGROUND
1- In cardiac ATTR amyloidosis, late planar images (2-3h p.i.) with [99mTc]Tc-DPD are standard, but earlier images (1h p.i.) are recommended with [99mTc]Tc-PYP.
2- Early and late images for [99mTc]Tc-DPD have not been directly compared.
3- In SPECT/CT, L-mode detector configuration (i.e., 90°angle) is recommended by consensus.
4- However, direct comparison with H-mode (180°angle) is missing.
Copyright American Society of Nuclear Cardiology

## Slide 3
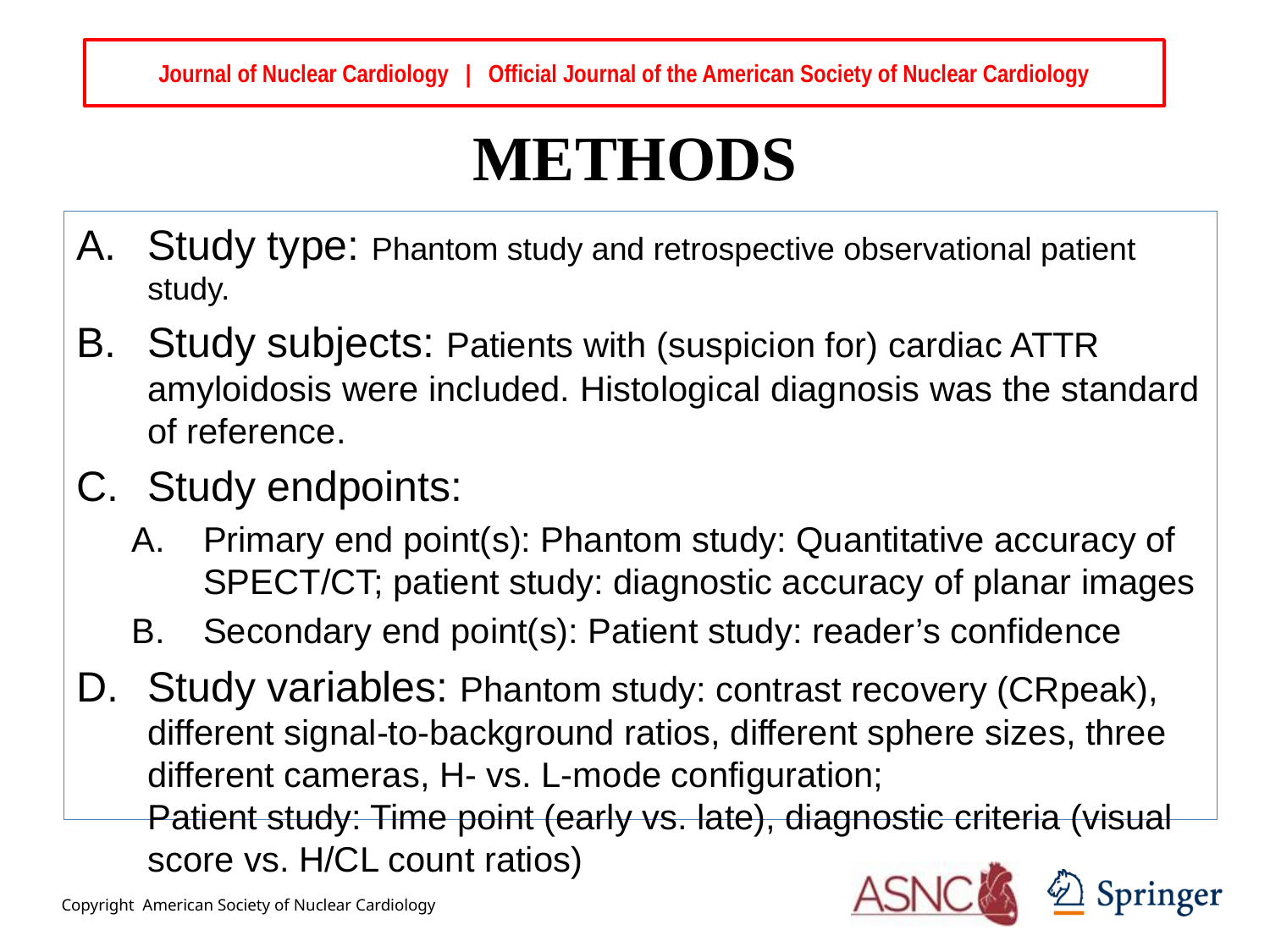

Journal of Nuclear Cardiology | Official Journal of the American Society of Nuclear Cardiology
# METHODS
Study type: Phantom study and retrospective observational patient study.
Study subjects: Patients with (suspicion for) cardiac ATTR amyloidosis were included. Histological diagnosis was the standard of reference.
Study endpoints:
Primary end point(s): Phantom study: Quantitative accuracy of SPECT/CT; patient study: diagnostic accuracy of planar images
Secondary end point(s): Patient study: reader’s confidence
Study variables: Phantom study: contrast recovery (CRpeak), different signal-to-background ratios, different sphere sizes, three different cameras, H- vs. L-mode configuration;
Patient study: Time point (early vs. late), diagnostic criteria (visual score vs. H/CL count ratios)
Copyright American Society of Nuclear Cardiology

## Slide 4
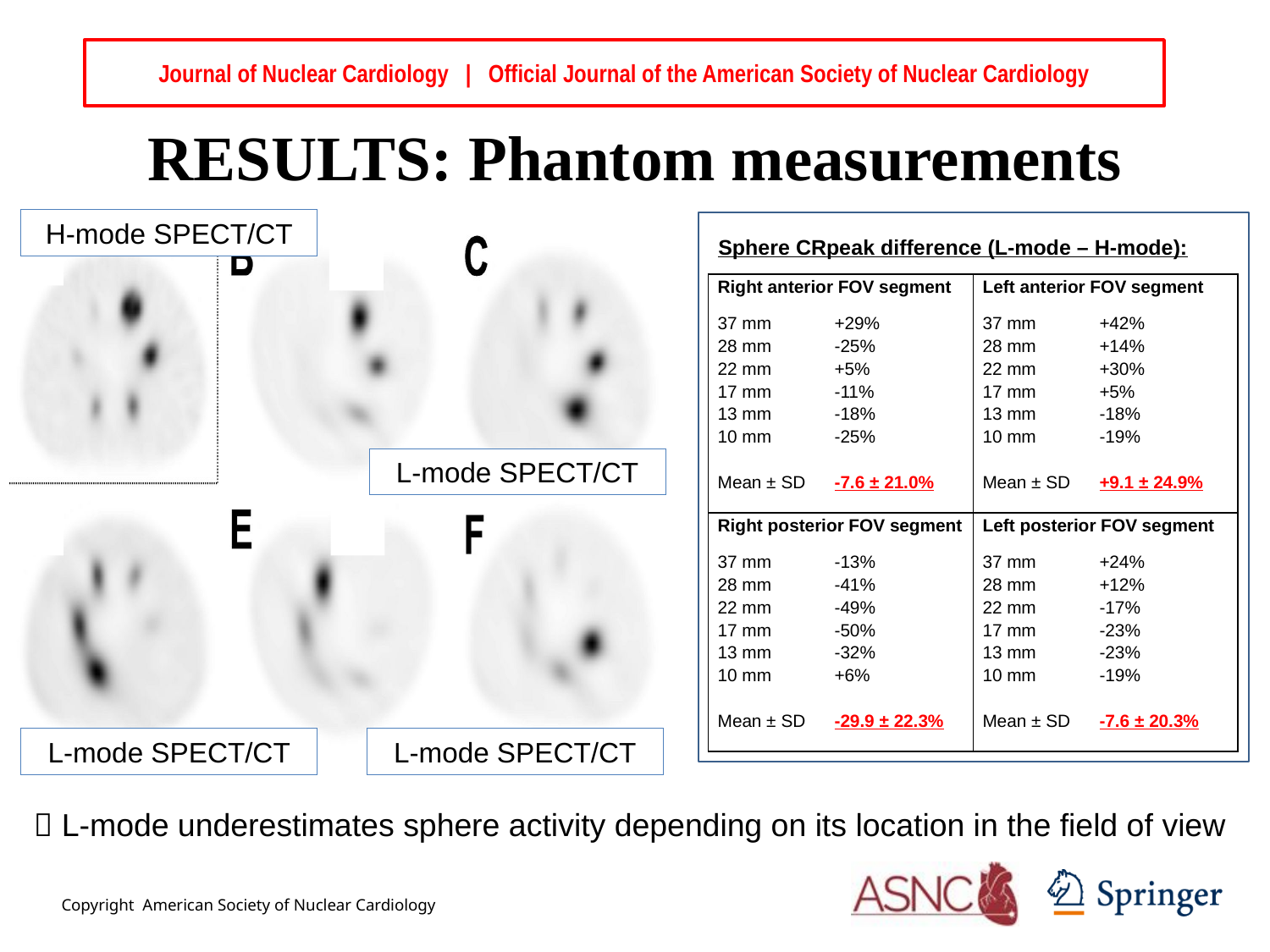

Journal of Nuclear Cardiology | Official Journal of the American Society of Nuclear Cardiology
# RESULTS: Phantom measurements
H-mode SPECT/CT
Sphere CRpeak difference (L-mode – H-mode):
| Right anterior FOV segment | | Left anterior FOV segment | |
| --- | --- | --- | --- |
| 37 mm 28 mm 22 mm 17 mm 13 mm 10 mm   Mean ± SD | +29% -25% +5% -11% -18% -25%   -7.6 ± 21.0% | 37 mm 28 mm 22 mm 17 mm 13 mm 10 mm   Mean ± SD | +42% +14% +30% +5% -18% -19%   +9.1 ± 24.9% |
| Right posterior FOV segment | | Left posterior FOV segment | |
| 37 mm 28 mm 22 mm 17 mm 13 mm 10 mm   Mean ± SD | -13% -41% -49% -50% -32% +6%   -29.9 ± 22.3% | 37 mm 28 mm 22 mm 17 mm 13 mm 10 mm   Mean ± SD | +24% +12% -17% -23% -23% -19%   -7.6 ± 20.3% |
L-mode SPECT/CT
L-mode SPECT/CT
L-mode SPECT/CT
 L-mode underestimates sphere activity depending on its location in the field of view
Copyright American Society of Nuclear Cardiology

## Slide 5
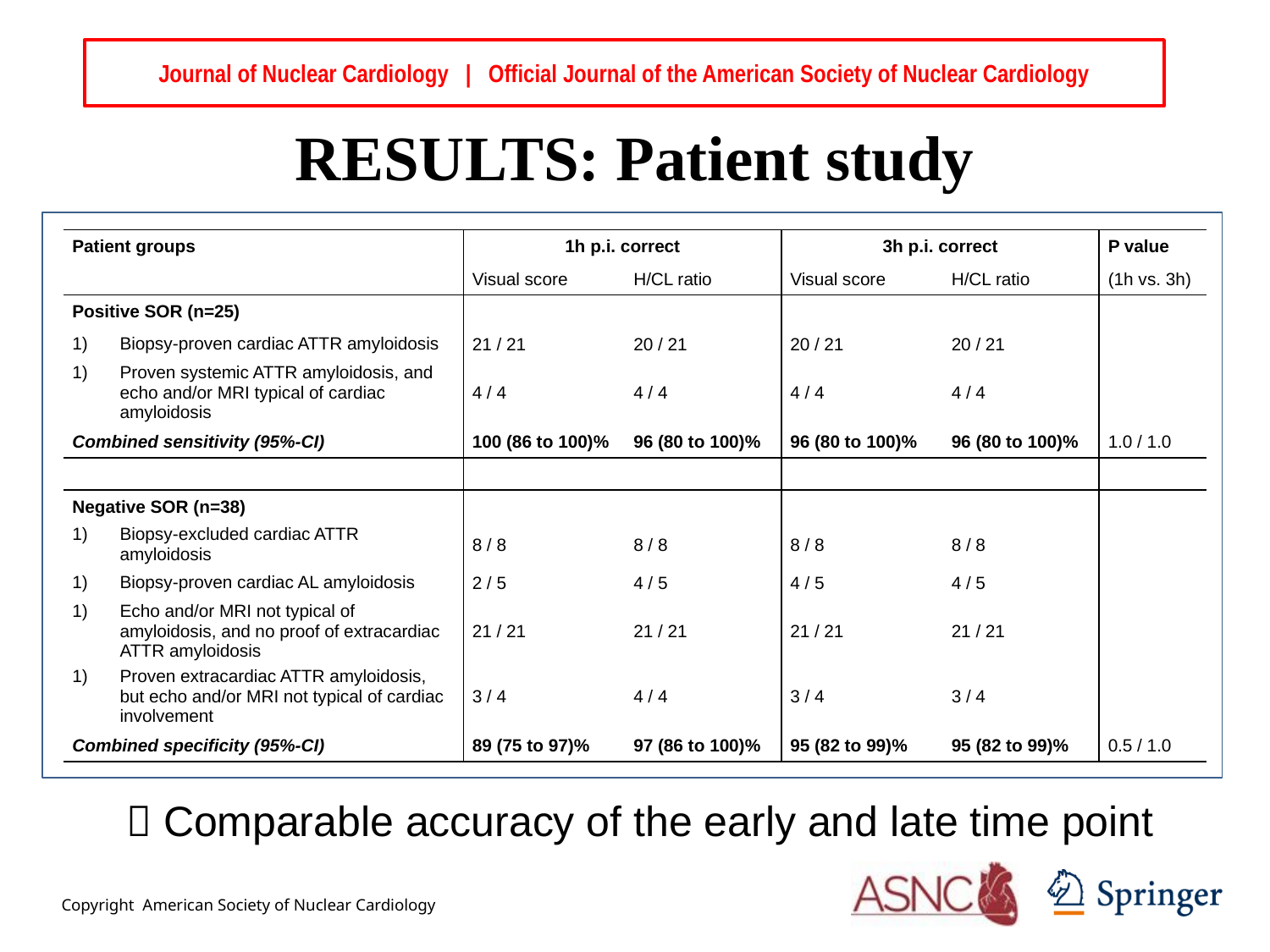

Journal of Nuclear Cardiology | Official Journal of the American Society of Nuclear Cardiology
# RESULTS: Patient study
| Patient groups | 1h p.i. correct | | 3h p.i. correct | | P value |
| --- | --- | --- | --- | --- | --- |
| | Visual score | H/CL ratio | Visual score | H/CL ratio | (1h vs. 3h) |
| Positive SOR (n=25) | | | | | |
| Biopsy-proven cardiac ATTR amyloidosis | 21 / 21 | 20 / 21 | 20 / 21 | 20 / 21 | |
| Proven systemic ATTR amyloidosis, and echo and/or MRI typical of cardiac amyloidosis | 4 / 4 | 4 / 4 | 4 / 4 | 4 / 4 | |
| Combined sensitivity (95%-CI) | 100 (86 to 100)% | 96 (80 to 100)% | 96 (80 to 100)% | 96 (80 to 100)% | 1.0 / 1.0 |
| | | | | | |
| Negative SOR (n=38) | | | | | |
| Biopsy-excluded cardiac ATTR amyloidosis | 8 / 8 | 8 / 8 | 8 / 8 | 8 / 8 | |
| Biopsy-proven cardiac AL amyloidosis | 2 / 5 | 4 / 5 | 4 / 5 | 4 / 5 | |
| Echo and/or MRI not typical of amyloidosis, and no proof of extracardiac ATTR amyloidosis | 21 / 21 | 21 / 21 | 21 / 21 | 21 / 21 | |
| Proven extracardiac ATTR amyloidosis, but echo and/or MRI not typical of cardiac involvement | 3 / 4 | 4 / 4 | 3 / 4 | 3 / 4 | |
| Combined specificity (95%-CI) | 89 (75 to 97)% | 97 (86 to 100)% | 95 (82 to 99)% | 95 (82 to 99)% | 0.5 / 1.0 |
 Comparable accuracy of the early and late time point
Copyright American Society of Nuclear Cardiology

## Slide 6
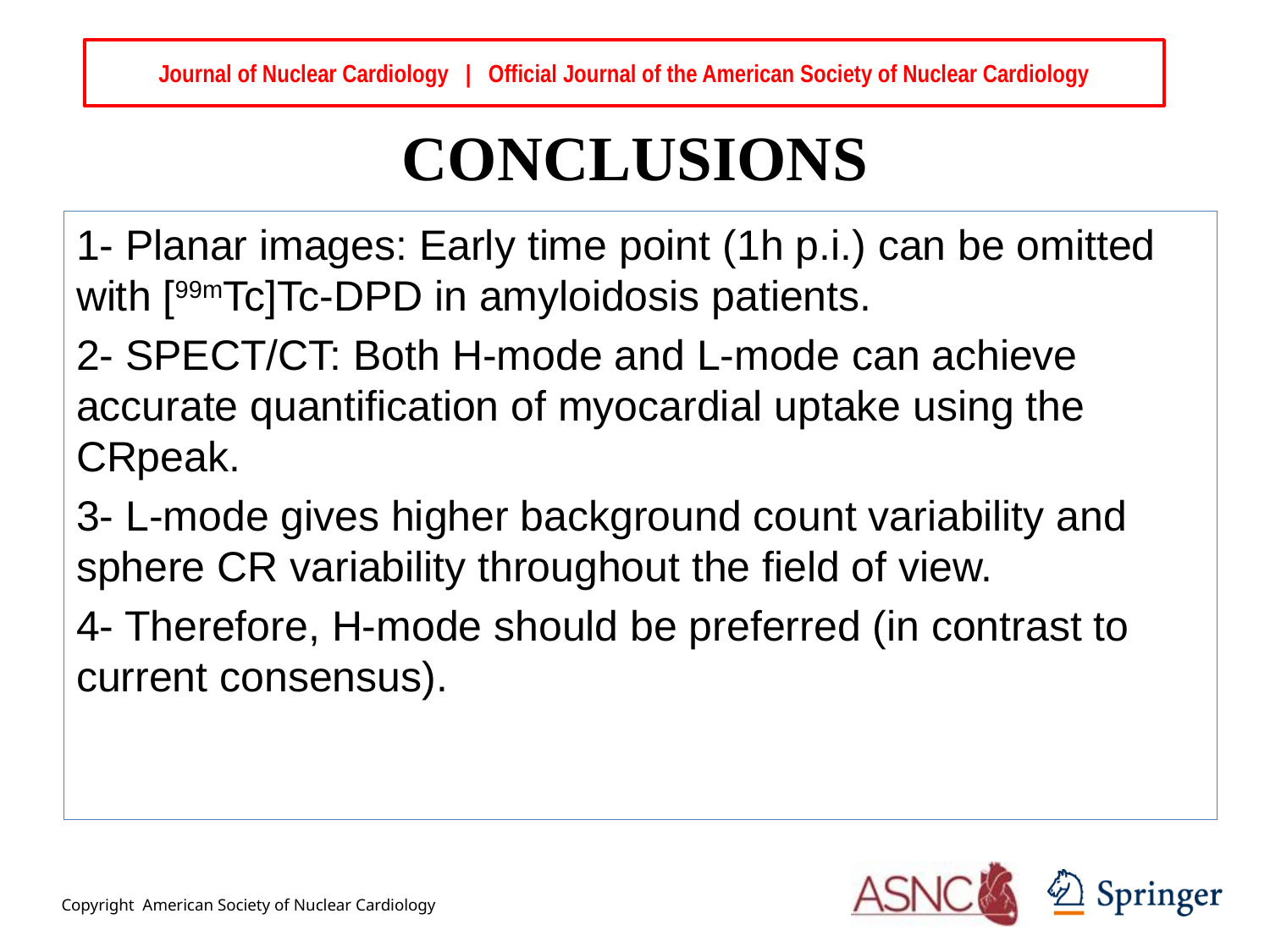

Journal of Nuclear Cardiology | Official Journal of the American Society of Nuclear Cardiology
# CONCLUSIONS
1- Planar images: Early time point (1h p.i.) can be omitted with [99mTc]Tc-DPD in amyloidosis patients.
2- SPECT/CT: Both H-mode and L-mode can achieve accurate quantification of myocardial uptake using the CRpeak.
3- L-mode gives higher background count variability and sphere CR variability throughout the field of view.
4- Therefore, H-mode should be preferred (in contrast to current consensus).
Copyright American Society of Nuclear Cardiology
